# Ecological Memory in Plants: Epigenetic Integration of Abiotic Stress and Climate Change

**DOI:** 10.3390/plants15040534

**Published:** 2026-02-08

**Authors:** Jun Zhang, Meng Song, Lu Zhang, Wenzhong Tian, Binbin Guo, Shuang Zhou, Chao Ma

**Affiliations:** 1 College of Agriculture, Henan University of Science and Technology, Luoyang 471023, China; zhangjun0105@126.com (J.Z.); m13072615152@163.com (M.S.); zhanglu@haust.edu.cn (L.Z.); guobin90@126.com (B.G.); zhoushuang2001@163.com (S.Z.); 2 Luoyang Academy of Agriculture and Forestry Sciences, Luoyang 471023, China; xxboy114@163.com

**Keywords:** epigenetics, DNA methylation, histone modification, non-coding RNA (ncRNA), environmental memory, epigenetic breeding

## Abstract

Against the backdrop of global climate change and the increasing frequency of extreme weather events, a central scientific question has emerged: how do plants adapt to such “pulsed” stressors? While traditional research has focused on immediate physiological responses and long-term genetic adaptation, this review introduces “ecological memory” as a novel integrative framework. It emphasizes the ability of plants to actively “record” past stress experiences through epigenetic mechanisms, thereby enhancing their adaptability to future adversities. This article systematically elucidates the molecular basis whereby abiotic stressors induce specific epigenetic modifications (e.g., DNA methylation and histone modifications) to form memories. It further discusses how such memories mediate physiological integration mechanisms, such as acclimation and priming-induced resistance at the individual level, and highlights potential pathways for transgenerational epigenetic memory transmission, which may accelerate population-level adaptive evolution. Finally, we evaluate the applications of the ecological memory concept in predicting species distribution, enhancing ecosystem resilience, and guiding the design of “climate smart” crops, aiming to shift the research paradigm from static tolerance studies to dynamic memory and adaptation frameworks.

## 1. Plant Epigenetics

Since its conceptual inception by Waddington [1], epigenetics has evolved from an abstract theory of “epigenesis” into a cutting-edge field of molecular biology. Compared to animals, plant epigenetics exhibits distinct characteristics: as sessile organisms, plants rely on high intracellular plasticity to adapt to fluctuating environmental stresses. Meanwhile, their differentiated somatic cells often retain totipotency, necessitating an epigenome that balances stable cellular identity with reprogramming potential [2]. These traits make plants ideal models for investigating the interplay between epigenetic regulation and environmental cues [3]. In recent years, advances in high-throughput sequencing technologies have expanded plant epigenetics research from model species (e.g., Arabidopsis) to major crops, uncovering critical roles in crop domestication and improvement [4].

## 2. Core Regulatory Mechanisms of Plant Epigenetics

### 2.1. DNA Methylation

**Figure 1:**
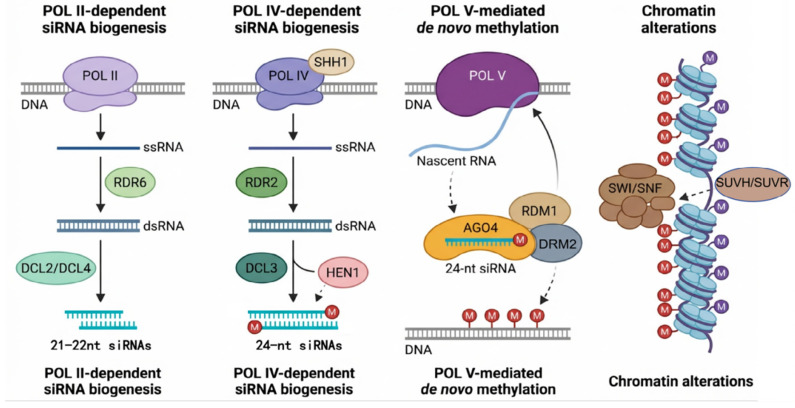
RNA-directed DNA methylation pathway in plants. The process involves Pol II- and Pol IV-dependent siRNA biogenesis (left and middle panels) and Pol V-mediated de novo methylation (right panel). The 24-nt siRNAs produced by RDR2 and DCL3 guide AGO4 to target loci transcribed by Pol V. The complex recruits *DRM2* for DNA methylation. This leads to chromatin alterations (far right) involving SUVH/SUVR histone methyltransferases and SWI/SNF remodeling complexes, establishing a repressive chromatin state.

DNA methylation represents the most extensively studied epigenetic marker in plants, primarily referring to the covalent modification of the fifth carbon atom of cytosine bases. As illustrated in Figure 1, the RNA-directed DNA methylation (RdDM) pathway is a key mechanism for establishing DNA methylation in plants. Plant DNA methylation exhibits unique sequence context patterns: CG, CHG, and CHH (where H = A, T, or C). The establishment and maintenance of methylation in these contexts are orchestrated by distinct enzyme systems: *MET1* is primarily responsible for maintaining CG methylation [5]; CMT3, and to some extent CMT2, maintain CHG and partial CHH methylation through interaction with histone marker H3K9me2 [6]; and *DRM2* is responsible for establishing methylation in all sequence contexts within the RdDM pathway [7,8]. Active removal of methyl groups, enabling dynamic regulation, is carried out by glycosylases such as *ROS1*, which initiate a base excision repair pathway [9,10]. The primary functions of DNA methylation include silencing transposable elements to maintain genome stability and playing crucial roles in genomic imprinting and gene expression regulation. Recent research further highlights its decisive role in plant cell differentiation, such as the specific differentiation of root hair and non-root hair cells, showcasing its importance beyond transposon silencing [11].

### 2.2. Histone Modification

**Figure 2:**
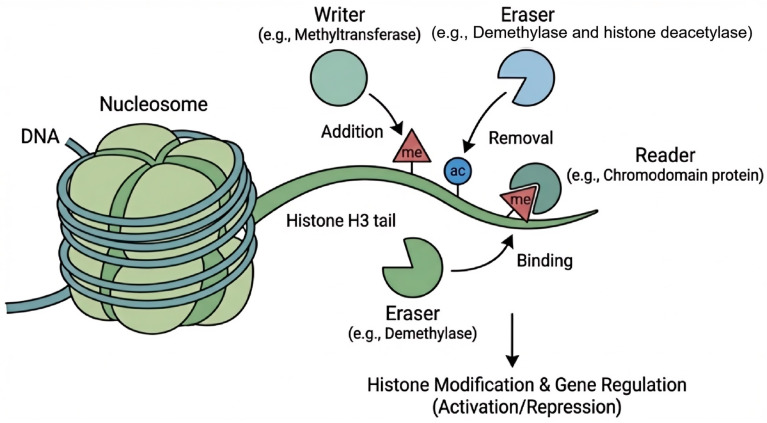
Schematic diagram of the histone modification pathway. The mechanism involves three classes of proteins acting on the N-terminal tail of histone H3 extending from the nucleosome core. “Writers” (e.g., histone methyltransferases) catalyze the addition of chemical groups, such as methyl groups (me), to specific amino acid residues. ”Erasers” (e.g., histone demethylases or deacetylases) are responsible for the removal of these marks (e.g., removing acetyl groups, ac, or methyl groups). “Readers” (e.g., proteins containing chromodomains) recognize and bind to specific modifications, translating the histone code into downstream biological outcomes, such as gene activation or repression.

The diverse covalent modifications on histone N-terminal tails constitute a complex “histone code”. Activating modifications, such as H3K4me3, H3K36me3, and H3K9ac, are typically enriched at transcription start sites and within actively transcribed gene bodies. In contrast, repressive marks like H3K9me2/3 and H3K27me3 are associated with constitutive and facultative heterochromatin, respectively [12,13]. In Arabidopsis, POLYCOMB REPRESSIVE COMPLEX 2 (PRC2) catalyzes the deposition of H3K27me3, which is critical for embryonic development, flowering time regulation (e.g., by repressing the floral inhibitor gene *FLC*), and organogenesis [14,15]. Meanwhile, H3K9me2 is often linked to transposon silencing and constitutive heterochromatin formation [16]. As illustrated in Figure 2, these modifications are dynamically regulated by specific “writers” (e.g., methyltransferases), “erasers” (e.g., demethylases and histone deacetylases), and “readers” (e.g., proteins containing chromodomains). For instance, histone deacetylases (HDACs) fine-tune rapid gene switching during plant stress responses [17].

### 2.3. Chromatin Remodeling and Non-Coding RNAs

**Figure 3:**
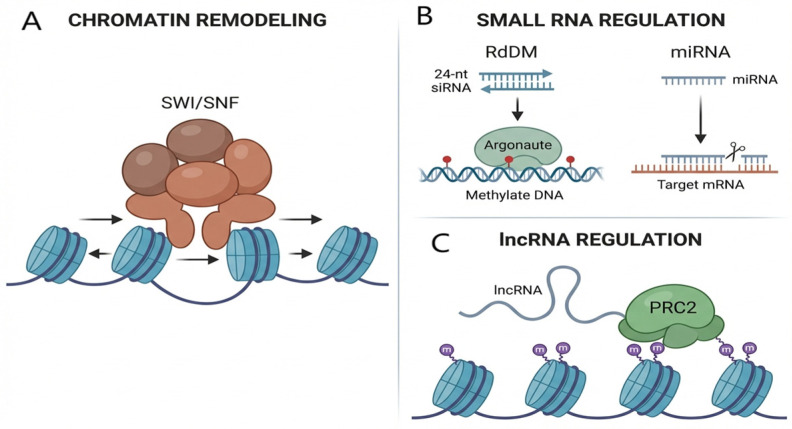
Schematic diagram of chromatin remodeling, non-coding RNA, and lncRNA regulatory mechanisms. (**A**) Chromatin remodeling: The SWI/SNF complex regulates DNA accessibility by altering nucleosome positioning (sliding/pelting). (**B**) Small RNA regulation: Left, the RdDM pathway, where 24-nt siRNA guides Argonaute proteins to methylate DNA; right, miRNA-mediated post-transcriptional gene silencing (targeted cleavage). (**C**) lncRNA regulation: lncRNAs recruit the PRC2 complex to specific chromatin sites, depositing the inhibitory H3K27me3 mark.

ATP-dependent chromatin remodeling complexes (e.g., the SWI/SNF family) modulate chromatin accessibility by sliding, ejecting, or replacing nucleosomes, thereby altering the binding interface for other regulatory factors [18,19]. Non-coding RNAs (ncRNAs) serve as key mediators of epigenetic regulation. As illustrated in Figure 3, 24-nt siRNAs direct *DRM2* to homologous DNA sequences for methylation via the RNA-directed DNA methylation (RdDM) pathway, representing a primary mechanism for viral defense and transposon silencing [7,20]. MicroRNAs (miRNAs), in contrast, function predominantly at the post-transcriptional level by mediating the cleavage or translational inhibition of target mRNAs [21]. Long non-coding RNAs (lncRNAs, such as *COOLAIR*) can interact with chromatin and recruit modifying complexes (e.g., PRC2 to the *FLC* locus), enabling spatiotemporal specific gene silencing [22,23].

### 2.4. Interplay Between Epigenetic Mechanisms

The aforementioned epigenetic mechanisms do not operate in isolation but are intricately interconnected, forming a sophisticated and interactive regulatory network. This crosstalk ensures precise gene control and enhances the system’s robustness and plasticity, allowing plants to integrate diverse developmental and environmental signals effectively [24].

#### 2.4.1. Positive Feedback Loop Between H3K9me2 and CHG Methylation

A classic example of direct epistasis is the reinforcement between the histone marker H3K9me2 and DNA methylation. The histone methyltransferases SUVH4/5/6 bind to methylated DNA (specifically mCHG and mCHH), which, in turn, catalyze the deposition of H3K9me2 [25]. This repressive histone marker then recruits the DNA methyltransferase *CMT3*, which specifically maintains CHG methylation [26,27]. This creates a self-reinforcing positive feedback loop that is critical for the stable silencing of transposable elements and the maintenance of constitutive heterochromatin [28].

#### 2.4.2. Integration by Multi-Functional Modules

Recent research has unveiled protein complexes that act as epigenetic integrators or hubs. A prime example is the CPL2-PHD2/3 module, which functions as a chromatin-responsive hub. This module can sense different chromatin contexts (e.g., H3K9me2 and H3K27me3) and recruit or cooperate with distinct effector pathways accordingly. It supports both the RdDM pathway and the SUVH4/5-CMT2 maintenance loop for CHH methylation, while also collaborating with the LHP1 protein to reinforce Polycomb-mediated gene silencing involving H3K27me3. This demonstrates how a single module can integrate signals from DNA methylation and histone modification pathways to produce a coherent silencing output, ensuring genomic integrity and developmental precision [29].

#### 2.4.3. Coordination in Gene Regulation

This interplay extends to the precise regulation of specific genes. For instance, long non-coding RNAs (lncRNAs) can recruit histone-modifying complexes like PRC2 (which deposits H3K27me3) to specific genomic loci, thereby leading to targeted gene silencing [23]. This mechanism is exemplified in the vernalization-mediated silencing of the *FLC* gene, where lncRNAs and chromatin remodeling cooperate to establish a stable repressed state. Furthermore, the activity of the RdDM pathway itself, responsible for generating siRNAs and initiating de novoDNA methylation, is influenced by the underlying chromatin state, highlighting the bidirectional relationship between chromatin structure and siRNA biogenesis [30,31].

This multilayered, interconnected network—characterized by mutual reinforcement, precise recruitment, and functional compensation—confers upon the plant epigenetics system remarkable robustness, stability, and adaptability. It enables plants to fine-tune gene expression dynamically in response to a complex array of internal and external cues.

## 3. Biological Functions of Plant Epigenetics

### 3.1. Precise Regulation of Growth and Development

**Figure 4:**
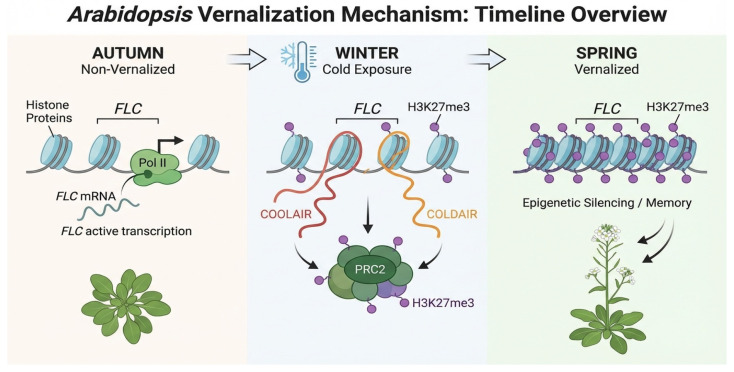
Epigenetic regulatory mechanisms during vernalization. The diagram illustrates the timeline of chromatin state changes at the *FLC* locus: In autumn, non-vernalized plants actively transcribe FLC via RNA Polymerase II (Pol II). The resulting mRNA inhibits flowering, leading to vegetative rosette growth. In winter, prolonged cold exposure induces the transcription of long non-coding RNAs (lncRNAs), COOLAIR (antisense) and COLDAIR (intronic). These lncRNAs facilitate the recruitment of the POLYCOMB REPRESSIVE COMPLEX 2 (PRC2) to the *FLC* locus, initiating the deposition of the repressive histone marker H3K27me3 (purple dots). In spring, when conditions return to warm temperatures, high levels of H3K27me3 are maintained across the *FLC* locus, establishing a stable “epigenetic memory” of winter. This leads to the permanent silencing of *FLC*, allowing the plant to transition from vegetative growth to flowering. Note: Similar epigenetic mechanisms, involving the silencing of DAM (Dormancy-Associated MADS-box) genes via H3K27me3, regulate bud dormancy release in perennial woody plants, functioning as a parallel form of winter memory.

A classic paradigm of epigenetic regulation in plant development is vernalization. As illustrated in Figure 4, during the overwintering period, low temperatures induce the expression of long non-coding RNAs (lncRNAs), including *COOLAIR* and *COLDAIR*, from the FLOWERING LOCUS C(*FLC*) locus. These lncRNAs recruit the Polycomb Repressive Complex 2 (PRC2), which catalyzes the deposition of the repressive histone marker H3K27me3 at the *FLC* locus. This leads to the epigenetic silencing of *FLC*, a central floral repressor, thereby promoting flowering in spring. This silenced state is mitotically stable, providing a “molecular memory” of winter duration that ensures flowering occurs under favorable conditions [15,22,23]. Beyond vernalization, epigenetic mechanisms play fundamental roles in diverse developmental processes, including seed development, dormancy, and germination [32], root patterning [11], and leaf senescence [33].

While vernalization controls flowering in herbaceous plants, perennial woody plants in temperate and boreal climates utilize a distinct but conceptually similar mechanism to manage winter survival: bud dormancy. Trees must “remember” exposure to chilling temperatures to track the passage of winter and time their bud break perfectly in spring.

Recent studies highlight the central role of Dormancy-Associated MADS-box (DAM) genes in this process, which function analogously to *FLC* in Arabidopsis. For example, in poplar (Populus) and peach (Prunus), *DAM* genes are expressed in autumn to induce and maintain dormancy. During prolonged cold exposure, epigenetic marks such as H3K27me3 progressively accumulate at *DAM* loci, eventually silencing them and allowing bud break when temperatures rise [34,35]. This “chilling requirement” acts as an ecological memory, preventing premature growth during transient warm spells in winter. The reversible nature of these markers ensures that the memory is reset annually, allowing trees to adapt to seasonal cycles over their long lifespan.

### 3.2. Genome Defense and Heterosis

DNA methylation and the RNA-directed DNA methylation (RdDM) pathway serve as a fundamental “immune system” in plants, effectively silencing transposable elements (TEs) and maintaining genomic integrity [7,8,24]. During events that cause significant genomic perturbation, such as polyploidization or wide hybridization, extensive epigenetic reprogramming occurs. This reprogramming can lead to the selective silencing of partial homoeologous genes, a phenomenon often described as “genomic shock” [36,37]. Such epigenetic restructuring is considered a critical source of gene dosage compensation as well as the emergence of novel phenotypes, including heterosis (hybrid vigor) [38].

For instance, studies on rice and wheat hybrids have shown that differential methylation of specific alleles contributes significantly to heterosis [39]. This suggests that the epigenetic landscape in hybrids, shaped by the interplay of parental genomes, can create optimal expression patterns for key growth- and yield-related genes. While DNA methylation patterns are altered in hybrids, research has demonstrated that the RdDM pathway itself, though responsible for establishing many of these methylation changes, is not the primary driver of heterosis. The chromatin remodeling factor *DDM1* has been identified as a key epigenetic regulator of heterosis, potentially by modulating the expression of genes involved in pathways like salicylic acid metabolism, rather than solely through DNA methylation changes [38,40].

### 3.3. Environmental Adaptation and Stress Memory

Plants can develop a faster and stronger response to a recurring stressor after initial exposure, a phenomenon known as stress memory, which is partly mediated by epigenetic mechanisms. For instance, following pathogen attacks in Arabidopsis, the level of H3K4me3—an activating histone marker—increased in response to promoters of defense-related genes (e.g., *WRKY* family genes). This elevated H3K4me3 level can be partially retained, maintaining these genes in a “primed” state and enabling their more rapid activation upon secondary infection, constituting a form of somatic memory [41,42]. Abiotic stresses such as drought, salinity, and extreme temperatures can also induce genome-wide epigenetic reprogramming, establishing stress memory that enhances tolerance to subsequent stress events [43].

The molecular basis of stress memory involves various epigenetic modifications, including DNA methylation, histone modifications (e.g., H3K4me3 and H3K27me3), and the action of small RNAs (Table 1) [41]. Some of these epigenetic marks, particularly certain DNA methylation variants, can potentially be transmitted through meiosis to offspring, even in the absence of the original stress signal, leading to transgenerational inheritance of adaptive traits [44,45]. However, the prevalence and adaptive significance of such transgenerational heritage in natural environments remain active areas of scientific debate. This multi-layered, interconnected network—characterized by mutual reinforcement, precise recruitment, and functional compensation—confers upon the plant epigenetic system remarkable robustness, stability, and adaptability. It enables plants to fine-tune gene expression dynamically in response to a complex array of internal and external cues [46,47].

## 4. From Static Endurance to Dynamic Memory: A New Paradigm for Plant Adaptation Under Climate Change

Current global change is characterized by gradual shifts in environmental factors to a pattern dominated by a significant increase in the frequency and intensity of extreme climate events, such as heatwaves, mega-droughts, and floods [61]. This “pulsed” stress poses severe challenges to plant survival, and the required adaptation strategies far exceed the slow pace of classical genetic adaptation. Traditional plant stress physiology has primarily elucidated the immediate pathways from stress perception and signal transduction to physiological and ecological responses [62], while evolutionary ecology has focused on the long-term genetic variation of adaptive traits. However, a critical knowledge gap exists between these two fields: can adaptive information acquired within an individual’s lifespan be stored, integrated, and influence future performance, or even be transmitted to offspring?

The concept of ecological memory provides a powerful framework to bridge this gap. It refers to the phenomenon whereby ecosystems (or their component species) are influenced by past disturbances or environmental conditions in a way that alters their current or future responses [63]. At the level of individual plants, this is essentially stress memory mediated by epigenetic modifications. Epigenetic mechanisms—such as DNA methylation, histone modifications, and small RNA regulation—can modulate gene expression without altering the DNA sequence, and maintain this modulation over time, even potentially transmitting it across generations [2,64]. Early conceptual explorations of “stress memory” in plants laid the groundwork for this paradigm [65]. To clarify the conceptual hierarchy and distinctions between these terms (e.g., priming and stress memory vs. ecological memory), we provide a detailed comparison in Table 2. We specifically focus on addressing the following three key questions. First, how abiotic stresses are encoded into epigenetic marks; second, how these marks form ecological memory at both individual and transgenerational levels; and third, how this memory influences multi-scale processes, from individual fitness to ecosystem dynamics. Through this lens, we seek to re-evaluate the adaptive potential of plants under climate change, offering new perspectives for predicting biodiversity changes and designing agricultural solutions.

## 5. The Molecular Core of Ecological Memory: Epigenetic Encoding of Abiotic Stress

### 5.1. Stress Signal Perception and the Initiation of Epigenetic Reprogramming

**Figure 5:**
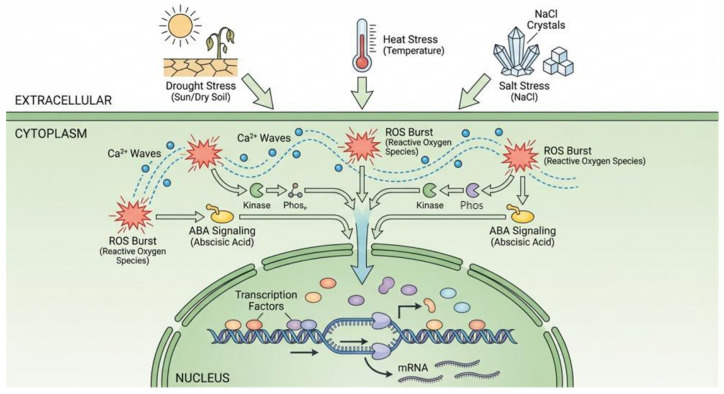
Perception of stress signals and initiation of epigenetic reprogramming. The diagram illustrates the cellular response mechanism to environmental stressors such as drought, heat, and salinity. Extracellular stress signals are perceived by the plant cell. (Middle) In the cytoplasm, signal transduction is mediated by second messengers, including calcium waves (Ca^2+^ Waves), reactive oxygen species bursts (ROS Burst), and abscisic acid signaling (ABA Signaling). These signals trigger protein kinase cascades, leading to the phosphorylation of downstream targets. Activated transcription factors enter the nucleus and bind to specific DNA regulatory elements, driving the transcription of stress-responsive genes (mRNA) to initiate physiological adaptation. Note: While not explicitly depicted as a separate icon, low-temperature stress activates similar signaling cascades (ROS, Ca^2+^, and ABA) to drought and salinity due to the physiological effects of freezing-induced cellular dehydration.

Upon perceiving stresses such as drought, high temperature, salinity, or low temperature, plants rapidly generate a complex signaling network involving calcium ions, reactive oxygen species (ROS), and plant hormones (e.g., abscisic acid and ABA), as illustrated in Figure 5. Notably, sub-zero temperatures induce cellular dehydration similar to drought and salt stress (via ice formation and decreasing water potential), thereby triggering overlapping signaling pathways. These early signals ultimately converge in the nucleus, triggering extensive transcriptional reprogramming. Recent research indicates that a key driver of this reprogramming is the dynamic remodeling of the epigenetic landscape [66,67]. For instance, drought stress can rapidly induce the eviction of the histone variant H2A.Z from the promoters of numerous stress-responsive genes, facilitating their swift activation [68]. This event marks the initial transition of stress signals from “transient cues” to a “latent memory”, representing the first step in encoding environmental experience into a stable molecular imprint.

### 5.2. Stress-Specific Epigenetic Marks

Different abiotic stresses can induce distinct and partially specific epigenetic signatures that serve as molecular hallmarks of stress memory. In Arabidopsis, dynamic changes in DNA methylation are a key carrier of stress memory. Drought stress, for instance, alters genome-wide DNA methylation patterns, with particularly significant and often persistent changes observed at CHH contexts (where H = A, T, or C), even after the stress is relieved [69]. Histone modifications also play a critical role. Activating markers associated with active transcription, such as H3K4me3 and H3K36me3, are maintained at the promoters of “memory genes” like RD29B and RAB18 following initial dehydration stress. This maintenance primes these genes for more rapid and robust activation upon subsequent stress encounters [65,70]. Conversely, salt stress can induce dynamic changes in the repressive marker H3K27me3 at specific gene loci, fine-tuning the expression of genes involved in ion homeostasis [52]. Furthermore, chromatin remodelers like *DDM1* are essential for facilitating the access of DNA methyltransferases to heterochromatic regions, thereby enabling the establishment of stress-induced DNA methylation patterns [71].

### 5.3. Mechanisms of Stress Memory “Storage” and “Recall”

**Figure 6:**
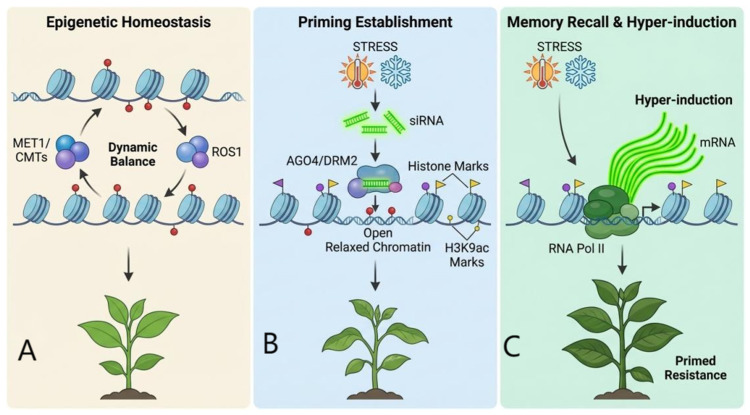
Molecular mechanisms for the stable maintenance of epigenetic marks. (**A**): Under normal conditions, a dynamic balance of DNA methylation is maintained by methyltransferases (*MET1*/CMTs) and demethylases (*ROS1*). (**B**): Upon initial stress exposure (e.g., heat or cold), stress-induced siRNAs and RdDM machinery (AGO4/*DRM2*) are activated. Concurrently, specific histone modifications (e.g., H3K9ac) are deposited, resulting in an “open” and relaxed chromatin state. This creates a “primed” memory. (**C**): Upon re-exposure to stress, pre-established open chromatin accessibility allows RNA Polymerase II (RNA Pol II) to rapidly access target genes, leading to transcriptional hyper-induction (massive mRNA production) and enhanced stress resistance.

The stable maintenance (“storage”) and functional activation (“recall”) of stress memory rely on specific molecular mechanisms. The persistence of DNA methylation marks is governed by a dynamic equilibrium between methyltransferases (e.g., *MET1*, CMTs, DRMs) and demethylases (e.g., *ROS1*, DME). Additionally, certain stress-induced small interfering RNAs (siRNAs) can guide the establishment and maintenance of DNA methylation at specific genomic loci, potentially providing a sequence-specific guide for memory [32]. When plants encounter a subsequent stress episode, as shown in Figure 6, these pre-established, permissive chromatin states—such as an open chromatin conformation and specific histone marks—enable the rapid recruitment of RNA Polymerase II, leading to the “hyper-induction” of target genes. This facilitated transcriptional response constitutes the molecular basis of primed resistance [41,72]. Beyond transcription, regulation at the level of RNA metabolism, including increased stability of stress-induced transcripts, is also considered an important layer of memory maintenance that contributes to accelerated response [73].

### 5.4. Fate of Epigenetic Memory During Recovery: Resetting vs. Retention

During the ecological recovery period (post-stress), the fate of epigenetic memory is determined by an antagonistic balance between active erasure and maintenance mechanisms.

Active Erasure (Resetting): To avoid the deleterious effects of constitutively active stress responses on plant growth/yield (the growth-defense trade-off), most stress-induced chromatin modifications are transient. For instance, the stress-responsive histone marker H3K27me3 is actively removed by specific histone demethylases (e.g., REF6 in Arabidopsis) to reset gene expression patterns. Similarly, active DNA demethylation, mediated by DNA glycosylases such as *ROS1* and DME, prevents the permanent accumulation of hypermethylation that could limit phenotypic plasticity [41,73].

Partial Retention (The “Poised” State): However, memory is not always completely erased. Certain “memory markers”, particularly H3K4me3 and specific DNA methylation patterns, can be partially retained at memory gene loci (e.g., RD29B, P5CS1) for days or even weeks after stress cessation. This partial retention does not sustain high-level transcription but maintains a distinct chromatin structure known as a “poised state”. This state keeps the nucleosomes accessible, allowing RNA Polymerase II to stall at the promoter (stalled Pol II), enabling a rapid transcriptional reboot if the stress recurs. Thus, while the physiological signal fades, the molecular footprint persists to confer future adaptive advantages [58].

## 6. Individual-Level Memory Manifestations: Priming, Acclimation, and Physiological Integration

### 6.1. Short-Term Memory and Priming (Stress Priming)

Plants pre-exposed to mild stress can exhibit a faster and more robust defense response upon encountering a subsequent, more severe stress: a phenomenon known as priming or acclimation. At the molecular level, this is attributed to latent transcriptional preparedness. For instance, in Arabidopsis thaliana, pre-treated with mild dehydration, the transcriptional activation of stress-responsive genes (e.g., RD29A and *COR15A*) during a second dehydration event occurs with significantly greater speed and amplitude compared to naive plants [70]. At the cellular level, guard cells can even demonstrate an independent transcriptional memory program during repeated exposure to dehydration [74]. Physiologically, this manifests as a quicker activation of antioxidant enzymes (e.g., SOD, CAT), a more rapid accumulation of osmolytes (e.g., proline, glycine betaine), and better protection of the photosynthetic apparatus [73].

### 6.2. Long-Term Memory and Structural Acclimation (Hardening)

Persistent or repeated stress exposure can lead to more enduring epigenetic states and physiological/structural alterations, a process often termed hardening. For example, plants repeatedly exposed to non-lethal low temperatures undergo stable changes in cell membrane lipid composition, the antioxidant system, and the basal expression levels of cold-responsive genes, thereby acquiring enhanced freezing tolerance [75]. In woody plants, trees that experienced drought in one growing season may maintain more conservative stomatal behavior (e.g., earlier closure and lower maximum conductance) in the subsequent season to reduce the risk of water stress [76]. Studies on key Mediterranean species like the holm oak (Quercus ilex) have shown that trees previously exposed to heat or drought stress maintain higher Photosystem II efficiency during later stress events, demonstrating cross-seasonal stress memory [77]. This structural acclimation involves more profound epigenetic and transcriptomic restructuring [78].

### 6.3. Costs and Trade-Offs of Memory

The establishment of ecological memory is not without costs. Maintaining specific epigenetic states, constitutively expressing certain defense-related genes, or altering morphological structures requires additional resources (carbon, nitrogen, and energy). This can lead to trade-offs between growth and reproduction under resource-limited conditions. For instance, primed plants often exhibit reduced growth rates during recovery phases compared to non-primed counterparts [79]. Such trade-offs form a crucial basis for natural selection acting on ecological memory, determining its adaptive value in specific environments. A complex balance exists between strength, duration, and cost of memory.

## 7. Transmission of Transgenerational Memory and Evolutionary Potential

### 7.1. The Flow of Epigenetic Information in the Reproductive System

The existence of transgenerational epigenetic memory challenges the conventional notion that acquired traits cannot be inherited. The key lies in whether stress-induced epigenetic marks can escape reprogramming during meiosis and fertilization or be re-established in the zygote [80]. In plants, because germline cells develop later, the epigenetic states experienced by somatic cells can directly influence the gametes or the early developing embryo. Studies have shown that parental exposure to stresses (e.g., pathogen infection, UV-C, and salinity) in Arabidopsis thaliana can lead to altered resistance, flowering time, and other traits in the offspring, even when they have not encountered the same stress [52,81].

### 7.2. Potential Transmission Mechanisms: siRNAs and Endosperm Programming

**Figure 7:**
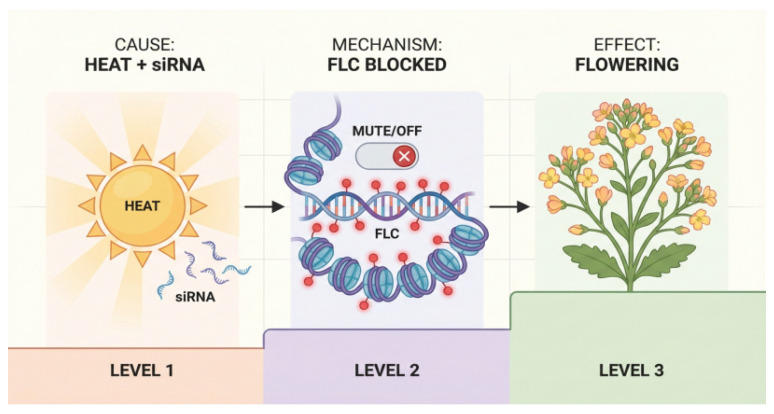
Schematic diagram of siRNA silencing the *FLC* gene affecting offspring flowering time. The process is divided into three levels, illustrating the cause-and-effect relationship: (Level 1) Heat stress induces the accumulation of specific siRNAs. (Level 2) These siRNAs target the flowering repressor gene *FLC*, leading to chromatin condensation and transcriptional silencing (depicted as “Mute/Off”). (Level 3) The repression of *FLC* relieves the inhibition on flowering, resulting in the transition to the reproductive stage (flowering) as a stress adaptation strategy.

Small interfering RNAs (siRNAs) are key candidates for transmitting transgenerational signals. Stress-induced siRNAs can move to reproductive cells and re-establish DNA methylation patterns in the offspring embryo via the RdDM pathway, thereby silencing specific genes [32]. As illustrated in Figure 7, heat stress-induced siRNAs can target flowering genes like *FLC*, influencing the flowering time of the progeny. Furthermore, the epigenetic programming of the endosperm (a triploid tissue) by parental stress may also impact embryonic development as the endosperm is a critical source of nutrients and signaling molecules for the embryo [81]. Parental stress might also shape offspring phenotypes through maternal effects, such as influencing seed size, hormone levels, and metabolite content [82].

### 7.3. Transgenerational Decay and Reversibility of Memory

A fundamental question in ecological epigenetics is the persistence of transgenerational memory. Unlike DNA mutations, which are stable and permanent, stress-induced epigenetic inheritance typically exhibits a phenomenon of “transgenerational washout” or decay.

Epigenetic memory serves as a “soft” adaptation or a “bethedging” strategy rather than a permanent fixation [83]. In the immediate progeny (F1) of stressed parents, memory effects (e.g., primed defense gene expression) are often strongest. However, if the offspring grow in a stress-free environment, the adaptive value of this memory decreases, and the associated metabolic costs (e.g., resource allocation and defense overgrowth) become maladaptive.

Empirical evidence suggests that without recurrent stress, transgenerational memory markers usually fade within one to three generations [45]. For example, hyperosmotic stress memory in Arabidopsis is robust in the F1 generation but largely reverts to the basal state by the F3 generation [52].

The mechanistic basis for this decay lies in the extensive epigenetic reprogramming that occurs during gametogenesis (meiosis) and early embryogenesis. Although some Differentially Methylated Regions (DMRs) escape this erasure and transmit memory, the efficiency of this escape decreases over generations without the reinforcement of the initial trigger (the stressor). The gradual activity of demethylases (e.g., *ROS1*) and the lack of small RNA reinforcement lead to the progressive loss of these epialleles, ensuring the population resets its phenotype to match the current stable environment [84].

### 7.4. Ecological and Evolutionary Implications

Transgenerational memory has the potential to accelerate microevolutionary adaptation in populations facing rapid environmental change. It provides a Lamarckian-like, additional source of adaptive phenotypic variation, enabling offspring to be “preadapted” to environments experienced by their parents [49]. This concept of “extended heredity” broadens our understanding of inheritance itself [85]. Empirical studies support this notion, demonstrating that epigenetic variation can contribute to fitness in Arabidopsis thaliana independently of DNA sequence variation [86] and that DNA methylation can mediate the inheritance of adaptive transgenerational plasticity [49].

However, the evolutionary significance of transgenerational memory remains a subject of active debate. Key points of contention revolve around the specificity of memory (how precisely it corresponds to particular stressors), its stability across generations, the number of generations through which memory persists, and its net adaptive value under fluctuating natural conditions, especially given the potential metabolic costs associated with maintaining a “primed” state.

A critical development in this field is to empirically determine the contribution of transgenerational epigenetic memory to population dynamics and the adjustment of species’ geographic range in wild plant populations [87]. Understanding how this mechanism influences species’ responses to contemporary climate change is a major focus of ongoing research.

## 8. From Individuals to Ecosystems: The Multi-Scale Impacts of Ecological Memory

While the molecular mechanisms described in previous sections are mechanistically supported (Evidence Level I), the ecological implications discussed below represent proposed hypotheses (Evidence Level III) and empirical observations that lack strict causal confirmation (Evidence Level II). The following sections explore these potential impacts based on current theoretical frameworks.

### 8.1. Shaping Population Dynamics and Genetic Structure

Individuals with different stress experiences (and thus different ecological memories) are hypothesized to exhibit variation in fitness components (survival, growth, and reproduction) in fluctuating environments. This potential variation within a population could theoretically alter its growth trajectory. In the long term, theoretical models suggest that if epigenetic variation is linked to genetic variation (e.g., via epialleles) or influences mating success, ecological memory might indirectly affect the population’s genetic structure [88]. The capacity for memory itself is proposed to be a selectable trait, subject to natural selection.

### 8.2. Influencing Community Composition and Species Coexistence

It is hypothesized that the ability to form and maintain ecological memory varies among species or genotypes. Under scenarios of increased stress frequency due to climate change, species with stronger memory capacities (e.g., those that can resist recurrent drought more effectively through priming) could theoretically gain a competitive advantage, potentially altering competitive balances and community composition [89]. Empirically, observations indicate that drought legacies have the potential to shift plant community composition and subsequent ecosystem functioning [90]. Thus, ecological memory is proposed to act as a crucial trait determining species coexistence and the maintenance of biodiversity, although causal links in complex communities remain to be fully established.

### 8.3. A Regulator of Ecosystem Function and Resilience

At the ecosystem scale, the memory effects of numerous individual plants are postulated to aggregate, potentially manifesting as changes in the distribution of functional traits (e.g., community-level mean stomatal conductance, water use efficiency, and root distribution). This, in turn, could influence key ecosystem processes such as carbon assimilation, evapotranspiration, and nutrient cycling. For example, a forest that has experienced past drought might adopt more conservative water-use strategies at the ecosystem level, hypothetically influencing watershed hydrology [91]. Field studies on Mediterranean ecosystems suggest that trees with prior experience of heat stress recover their carbon sink function more effectively [77]. Consequently, ecological memory may represent an intrinsic determinant of ecosystem resistance and resilience (the capacity to recover from disturbances). Quantifying ecological memory is, therefore, proposed as a key step to understanding and predicting ecosystem dynamics [62].

## 9. Application Prospects, Key Technologies, and Future Challenges

### 9.1. Designing “Climate-Smart” Crops and Guiding Ecological Restoration and Biodiversity Conservation

The principles of ecological memory offer novel strategies for crop breeding and cultivation management. By employing molecular marker-assisted selection or gene editing technologies, crop varieties with stronger and more precise stress memory capabilities can be selected or developed. For instance, varieties that can “remember” early drought and activate efficient water-saving mechanisms during later critical growth stages have been explored [72]. In forestry, selecting provenances with strong potential for ecological memory can enhance the success rate of afforestation under climate change [78]. Furthermore, applying mild stress “hardening” treatments during the seedling stage represents a low-cost and sustainable agronomic measure to improve crop resilience in the field. Epigenetics adds a new dimension to crop improvement. Epigenetic markers (epimarks) can complement traditional molecular markers in predicting agronomic traits [88]. Epigenetic editing technologies, such as fusing Cas9 with epigenetic modifiers (e.g., TET1 and p300), enable precise editing of DNA methylation or histone modifications at specific gene loci without altering the DNA sequence, paving the way for creating safe and reversible germplasm resources [92,93]. The use of chemical agents (e.g., demethylating agents) to gently induce epigenetic variations also holds promise for enhancing crop tolerance to environmental stresses [94]. In crops like rice and tomato, several important agronomic traits controlled by epigenetic variation have been identified, providing direct targets for epigenetic breeding [95,96].

In ecological restoration, priority should be given to using provenances with local adaptation and those likely carrying beneficial stress memories for seedling cultivation. In conservation biology, understanding the ecological memory potential of species helps to more accurately predict the responses of vulnerable species to climate change and identify populations that may require assisted migration [49]. Incorporating ecological memory parameters into species distribution models can enhance the accuracy of predictions.

### 9.2. Application Potential: Epigenetic Breeding for Stress Tolerance

The transgenerational inheritance of epigenetic modifications offers a novel pathway for crop breeding, distinct from traditional genetic breeding. This approach, termed “Epigenetic Breeding,” aims to utilize stable epialleles to develop germplasm resources with enhanced stress tolerance [97].

While single-generation stress memory may fade (as discussed in Section 7.3), successive generations of induced treatments can reinforce these epigenetic marks. Evidence suggests that recurrent exposure to specific stressors (e.g., cold or drought) over multiple generations can lead to the “fixation” of favorable chromatin states. This process converts transient stress responses into stable heritable traits, effectively “training” the plant genome [98].

For instance, by subjecting populations to repeated cold stress selection, breeders can select individuals with constitutively activated defense pathways (e.g., higher basal expression of COR genes) driven by DNA hypomethylation variants. These selected populations, known as Epilines or Epigenetic Recombinant Inbred Lines (epiRILs), exhibit phenotypic diversity and improved resilience even in the absence of genetic variation [99].

Unlike genetic mutation breeding, which is random and slow, epigenetic breeding allows for the rapid generation of phenotypic variations specifically targeted toward adaptation to environmental shifts. This strategy is particularly valuable for improving complex traits such as drought resistance and yield stability under climate change [100].

### 9.3. Key Technologies

The high-throughput sequencing revolution has propelled the advancement of epigenomics. Whole-genome bisulfite sequencing (WGBS) generates single-base resolution DNA methylation maps [101]; Chromatin Immunoprecipitation sequencing (ChIP-seq) analyzes the genome-wide distribution of histone modifications and transcription factors [102]; Assay for Transposase-Accessible Chromatin using sequencing (ATAC-seq) reveals open chromatin regions [103]. Integrated multiomics analysis of these data is a core approach for deciphering epigenetic regulatory networks. The emergence of single-cell epigenomics technologies, such as single-cell ATAC-seq (scATAC-seq), is beginning to unveil the cell-type-specific epigenetic landscape within plant tissues [104].

### 9.4. Core Scientific Challenges

It is critical to recognize the heterogeneity in evidence quality across different scales of ecological memory research. Current findings can be stratified into three levels of certainty. First, at the molecular and individual level (Level I), evidence is robust and causal. Studies using model organisms (e.g., Arabidopsis) under controlled laboratory conditions have linked specific epigenetic mechanisms (e.g., DNA methylation, histone modifications like H3K4me3) to stress memory phenotypes [41]. Second, at the transgenerational level (Level II), evidence is moderate. While heritable phenotypic changes are frequently observed in crops and greenhouse experiments, strictly distinguishing “pure” epigenetic inheritance from maternal effects (e.g., seed nutrient provision) or genetic selection remains methodologically challenging, especially in non-model species [64,105]. Third, at the ecosystem scale (Level III), implications are largely inferential. Claims regarding the role of epigenetic memory in stabilizing community structure or facilitating shifts in species range are predominantly based on theoretical models or extrapolations from individual-level data [83]. While promising, these ecosystem-level effects currently lack long-term, causal empirical validation in complex natural settings. Therefore, caution is warranted when scaling up molecular mechanisms to predict ecosystem dynamics.

The field still faces numerous challenges. First, when establishing causal links in complex environments, it is difficult to strictly distinguish the effects of epigenetic memory from other factors (e.g., subtle physiological changes, soil microbiome legacy, and maternal resource effects) on offspring phenotypes. Second, stability and reversibility of memory should be considered: How long can memory last? Under what conditions is it erased? What is the persistence of the associated epigenetic markers? Third, most evidence of prevalence and adaptiveness in natural populations comes from laboratory model plants under controlled stresses. The importance, adaptive value, and costs of ecological memory in complex, fluctuating natural communities with multiple concurrent stresses urgently require validation [87].

### 9.5. Future Directions

Future research should also address the following issues. First, integrated multiomics (epigenomics, transcriptomics, metabolomics, proteomics) combined with genetic approaches should be used to dissect the complete causal pathway of memory formation, storage, and recall. Second, the ecological and evolutionary consequences of memory, and its variation within and between species, should be quantified through long-term field-controlled experiments and natural gradient surveys. Third, theoretical models and predictive frameworks should be developed that integrate epigenetic processes, phenotypic plasticity, and population dynamics to enhance the predictive capability for biosphere responses to climate change. Fourth Memory “reset” mechanisms and strategies should be explored to optimize memory and maximize adaptive benefits while minimizing growth costs.

## 10. Summary

The concept of ecological memory elevates the response to abiotic stress from an instantaneous physiological reaction to a dynamic adaptive process spanning temporal scales (from minutes to generations) and spatial scales (from molecular to ecosystems). Centered on epigenetic mechanisms, ecological memory represents an active, proactive strategy for plants to cope with uncertain environments under climate change. It not only transmits adaptive information within and across generations but may also provide feedback on ecosystem functions and climate systems by influencing population and community dynamics. Although significant challenges remain in mechanistic understanding, ecological relevance, and evolutionary significance, this field is developing rapidly. Future research integrating molecular biology, ecology, evolutionary biology, and climate change science will deepen our fundamental understanding of life environment interactions and provide nature-based innovative solutions for global change challenges.

## Figures and Tables

**Table 1:** Epigenetic modifications involved in various stress memories.

Modification Method	Types of Stress	Related Genes or Regulatory Factors	Species	Mechanism
DNA methylation	drought		*Persicaria persicaria*	DNA methylation can mediate the maintenance of drought stress memory, while DNA demethylation treatment can remove drought stress memory [48,49].
			*Arabidopsis thaliana*	Drought stress memory leads to genome-wide DNA hypomethylation [50].
	salt		*Oryza sativa*	Salt stress leads to heritable DNA methylation changes [51]
		DNA glycosylase	*Arabidopsis thaliana*	Hypermethylation of specific DNA regions mediates salt stress memory, while DNA glycosylase can inhibit the inheritance of salt stress memory [52].
	organism	*PR-1*	*Arabidopsis thaliana*	The hypomethylation level of DNA enhances the expression of *PR-1* and mediates the inheritance of biotic stress memory [53].
histone modification	elevated temperature	*HSFA2*	*Arabidopsis thaliana*	*HSFA2* can maintain high levels of H3K4me3/2, thereby sustaining heat stress memory [54,55].
		FGT1	*Arabidopsis thaliana*	FGT1 induces and maintains the expression of memory genes and mediates stress memory by regulating nucleosome occupancy [56].
		*HSFA2*, REF6	*Arabidopsis thaliana*	*HSFA2* forms a positive feedback loop with REF6, maintaining heat stress memory regulated by H3K27me3 demethylation [57].
	drought	*RD29B*, *RAB18*	*Arabidopsis thaliana*	High levels of H3K4me3 modification can accelerate the transcription of *RD29B* and *RAB18* genes [58].
	salt	*HKT1*	*Arabidopsis thaliana*	A decrease in the level of H3K27me3 modification accelerates the transcription of the *HKT1* gene [43].
		*P5CS1*	*Arabidopsis thaliana*	High levels of H3K4me3 modification accelerate the transcription of the *P5CS1* gene [59].
	low temperature	*COR15A*, *ATGOLS3*	*Arabidopsis thaliana*	Decreased H3K27me3 modification promotes the transcription of *COR15A* and *ATGOLS3* genes [60].
		*FLC*	*Arabidopsis thaliana*	H3K27me3 interacts with the Polycomb Repressive Complex 2 (PRC2) in the promoter region of the *FLC* gene, thereby maintaining the repression of *FLC* gene expression [14].
	organism	*WRKY6*, *WRKY53*	*Arabidopsis thaliana*	H3K4me3 modification promotes the transcription of *WRKY6* and *WRKY53* [42].
		*PLANT DEFENSIN1.2*	*Arabidopsis thaliana*	H3K27me3 modification represses the transcription of the *PLANT DEFENSIN 1.2* gene [53].
		*PATHOGENESIS-RELATED GENE1*, *WRK-Y6*, *WRKY53*	*Arabidopsis thaliana*	H3K9ac modification promotes the transcription of *PATHOGENESIS-RELATED GENE 1*, *WRKY6* and *WRKY53* [53].

**Table 2:** Conceptual framework: definitions and hierarchies of memory-related terms.

Term	Conceptual Level	Definition	Operational Criteria and Key Features
Acclimation/Hardening	Organismal/Physiological	The adjustment of physiology or morphology in response to environmental change, increasing tolerance to subsequent stress. “Hardening” often refers specifically to pre-exposure to mild stress (e.g., cold hardening).	Design: Single or continuous stress exposure. Duration: Generally persists as long as the stress or season lasts. Readout: Immediate survival rate, electrolyte leakage, and photosynthesis rate.
Priming	Molecular/Cellular	A physiological state induced by a transient stimulus (priming cue) that enables the plant to respond more rapidly and/or robustly to a future stress. It is the mechanism underlying somatic memory.	Design: Requires Stress–Recovery–Rechallenge design. Cost: Low cost in the absence of stress (latent state). Readout: Potentiated gene expression (e.g., WRKYs), accumulation of signaling metabolites.
Stress Memory (Somatic)	Organismal	The retention of information from a past stress event after the cue has ceased, modifying the phenotype upon re-exposure within the same generation.	Duration: Days to weeks (short-term) or months (long-term). Reversibility: Often reversible (can be reset). Mechanism: Epigenetic marks (DNA methylation, H3K4me3), protein stability.
Transgenerational Memory	Population (Inter-generational)	The transmission of stress-induced phenotypic or epigenetic changes to offspring (F1, F2, etc.) that were not directly exposed to the initial stress.	Design: Stress on Parent (P0) → Assessment of Offspring (F1/F2) in control/stress conditions. Key: Must rule out direct embryo exposure (for true transgenerational inheritance).
Ecological Memory	Ecological (Community/Ecosystem)	The capacity of an ecosystem or community to retain information about past events (disturbances) and use it to shape current structure and future responses.	Scale: Large spatial/temporal scales. Components: Includes both biological memory (seeds, bud banks) and material memory (soil legacy, litter). Readout: Community composition, resilience, recovery speed.
Legacy Effects	Ecological (Soil–Plant Feedback)	The persistence of abiotic or biotic changes in the environment (often soil) caused by a previous plant or event, which influences subsequent plants.	Mechanism: Indirect mediation (e.g., altered soil microbiome, nutrient depletion, allelopathy). Distinction: Focuses on the environmental footprint rather than the organism’s internal state.

## Data Availability

No new data were created or analyzed in this study. Data sharing is not applicable to this article. This review does not contain any original data and is based entirely on information presented in previously published studies.

## Abbreviations

Abbreviation	Full Name	Description/Function
Enzymes and Protein Complexes		
RdDM	RNA directed DNA methylation	Epigenetic pathway involving small RNAs guiding DNA methylation.
Pol IV/V	RNA POLYMERASE IV/V	Plant-specific RNA polymerases essential for siRNA biogenesis and scaffold transcription.
RDR2/6	RNA-DEPENDENT RNA POLYMERASE 2/6	Enzymes that convert single-stranded RNA into double-stranded RNA.
DCL2/3/4	DICER LIKE 2/3/4	Ribonucleases that process dsRNA into 21–24 nt siRNAs.
AGO4	ARGONAUTE 4	Effector protein that binds siRNAs to target homologous DNA sequences.
*DRM2*	DOMAINS REARRANGED METHYLTRANSFERASE 2	The major de novo DNA methyltransferase in plants.
*MET1*	METHYLTRANSFERASE 1	Maintains CG DNA methylation during replication.
CMT2/3	CHROMOMETHYLASE 2/3	Maintain non-CG (CHG and CHH) DNA methylation.
*ROS1*	REPRESSOR OF SILENCING 1	A DNA glycosylase that actively removes DNA methylation (demethylation).
SUVH/SUVR	SUPPRESSOR OF VARIEGATION 3-9 HOMOLOG/RELATED	Histone methyltransferases responsible for H3K9 methylation.
PRC2	POLYCOMB REPRESSIVE COMPLEX 2	A complex that deposits H3K27me3 marks to silence genes (e.g., *FLC*).
SHH1	SAWADEE HOMEODOMAIN HOMOLOG 1	An accessory protein linking H3K9me2 marks to Pol IV recruitment.
SWI/SNF	SWITCH/SUCROSE NON-FERMENTABLE	An ATP-dependent chromatin remodeling complex.
HEN1	HUA ENHANCER 1	A methyltransferase that stabilizes siRNAs by methylating their 3′ ends.
RDM1	RNA-DIRECTED DNA METHYLATION 1	A protein bridging AGO4 and *DRM2*.
Epigenetic Marks and Modifications		
H3K4me3	Histone H3 Lysine 4 trimethylation	A histone marker associated with active gene transcription.
H3K9me2	Histone H3 Lysine 9 dimethylation	A repressive histone marker associated with heterochromatin.
H3K27me3	Histone H3 Lysine 27 trimethylation	A repressive marker mediating long-term gene silencing (e.g., in vernalization).
H3K9ac	Histone H3 Lysine 9 acetylation	A histone marker associated with open chromatin and gene activation.
Ph	Phosphate/Phosphorylation	The addition of a phosphate group to a molecule (e.g., protein activation).
Signaling and Genes		
ROS	Reactive Oxygen Species	Chemical species (e.g., H_2_O_2_) acting as signaling molecules under stress.
ABA	Abscisic Acid	A key plant hormone involved in stress responses.
*FLC*	FLOWERING LOCUS C	A key repressor gene of flowering in Arabidopsis.
siRNA	Small interfering RNA	Short, double stranded RNA molecules that guide gene silencing.
lncRNA	Long non-coding RNA	RNA molecules longer than 200 nucleotides involved in gene regulation.
